# Assessment of Bone Mass and Fracture Risk Using Trabecular Bone Score in Children with Autoimmune Gastrointestinal Diseases

**DOI:** 10.3390/nu18152454

**Published:** 2026-07-27

**Authors:** Anna Łupińska, Sara Aszkiełowicz, Arkadiusz Zygmunt, Renata Stawerska

**Affiliations:** 1 Department of Endocrinology and Metabolic Diseases, Polish Mother’s Memorial Hospital—Research Institute, 93-338 Lodz, Poland; sara.aszkielowicz@iczmp.edu.pl (S.A.); arkadiusz.zygmunt@umed.lodz.pl (A.Z.); renata.stawerska@umed.lodz.pl (R.S.); 2 Department of Developmental Age and Adult Endocrinology, Medical University of Lodz, 93-338 Lodz, Poland

**Keywords:** trabecular bone score, bone mineral density, autoimmune gastrointestinal disease, Crohn’s disease, celiac disease, pediatric osteoporosis, bone microarchitecture, children, vitamin D, body composition

## Abstract

**Background/Objectives**: Children with autoimmune gastrointestinal diseases are at increased risk of impaired bone health due to chronic inflammation, nutritional deficiencies, growth disturbances, and treatment-related factors. While dual-energy X-ray absorptiometry (DXA) is the standard method for assessing bone mineral density (BMD), it provides limited information on bone microarchitecture. The trabecular bone score (TBS), derived from lumbar spine DXA images, has emerged as a complementary marker of bone quality. This study aimed to evaluate bone mass and TBS in children with autoimmune gastrointestinal diseases and to assess the clinical utility of TBS in comparison with children with a history of fractures and healthy controls. **Methods**: This study included 152 children aged 5–18 years: 45 with autoimmune gastrointestinal diseases (Crohn’s disease, ulcerative colitis, or celiac disease), 37 with a history of fractures, and 70 healthy controls. Anthropometric measurements, serum 25-hydroxyvitamin D [25(OH)D] concentrations, DXA-derived parameters, and TBS values were analyzed. Bone mineral density was assessed at the lumbar spine and total body less head (TBLH), with additional adjustment for height-for-age Z-score (HAZ). TBS values were expressed as sex- and pubertal stage-adjusted Z-scores. **Results**: Low bone mass (aBMD_for age_ Z-score ≤ −2) was observed in 30.3% of participants at TBLH and 11.1% at the lumbar spine, whereas a TBS Z-score ≤ −2 was identified in 5.2% of children. No significant differences in TBS or TBS Z-scores were found among the study groups. In multivariable analysis, fracture history was independently associated with lower absolute TBS, whereas no independent predictors of TBS Z-score were identified. Children with fractures had significantly lower HAZ-adjusted lumbar spine aBMD Z-scores than children with autoimmune gastrointestinal diseases and controls. TBS Z-scores correlated positively with age-adjusted and HAZ-adjusted aBMD values but showed no association with BMI or serum 25(OH)D concentrations. **Conclusions**: In this cross-sectional study, TBS did not distinguish children with autoimmune gastrointestinal diseases from those with fractures or healthy controls in the unadjusted analyses. Although TBS was associated with selected DXA-derived measures of bone mineral density and fracture history was independently associated with lower absolute TBS after multivariable adjustment, no independent predictors of TBS Z-score were identified. These findings suggest that the clinical role of TBS in the assessment of pediatric bone health remains to be established. Larger prospective studies are crucial to determine whether TBS provides clinically meaningful information complementary to conventional DXA for the assessment of skeletal health and fracture risk in children. Larger prospective studies are needed to clarify the clinical value of TBS for fracture risk assessment in pediatric autoimmune gastrointestinal diseases.

## 1. Introduction

Dual-energy X-ray absorptiometry (DXA) remains the primary clinical modality for assessing bone health in both pediatric and adult populations. Areal bone mineral density (aBMD), derived from DXA, is strongly associated with fracture risk across all age groups and represents a key element in the diagnosis of osteoporosis in adults. However, bone strength and fracture susceptibility are determined not only by bone density but also by bone structure and quality. In recent years, the trabecular bone score (TBS), obtained from lumbar spine DXA images, has emerged as a complementary parameter that provides additional insight into skeletal microarchitecture [1]. TBS is based on texture analysis of gray-level variations within DXA images and serves as an indirect index of trabecular microstructure. Consequently, it offers a distinct advantage by detecting alterations in bone quality that are not captured by aBMD alone, particularly in conditions in which bone fragility is driven by impaired microarchitecture rather than reduced bone mass [2,3].

In adults, TBS has been shown to enhance fracture risk prediction when used in con-junction with aBMD [3,4,5]. However, its application in pediatric populations remains limited, largely due to the lack of age-, sex-, and pubertal stage-specific reference values, as well as the potential confounding effect of body composition, including abdominal adiposity [6,7].

Autoimmune gastrointestinal diseases, particularly inflammatory bowel disease (IBD), commonly manifest during childhood and adolescence, a critical period for skeletal acquisition and bone mass accrual [8,9]. As a result, children with IBD frequently exhibit impaired bone health compared with healthy peers, with deficits that may already be pre-sent at diagnosis [2,8]. A comparable pattern has been reported in celiac disease [10,11]. The underlying pathophysiology is multifactorial and includes chronic systemic inflammation, delayed linear growth and pubertal maturation, reduced lean body mass, and exposure to glucocorticoid treatment therapy [12,13]. Delayed growth and pubertal maturation observed in these children are attributed to multiple factors, including the underlying disease itself, disease-associated nutritional deficiencies, the often severe clinical course, and recurrent hospitalizations. Together, these factors may adversely affect somatic growth and the timing of pubertal development. Perturbations occurring during adolescence—a key window for attainment of peak bone mass—may have early and late effects consequences, including an increased risk of osteoporosis in later life [14].

Although DXA remains the standard method for assessing bone mineral density in pediatric patients, its interpretation is limited by the influence of body size and its inability to differentiate between cortical and trabecular bone compartments [15]. In this context, TBS represents a promising adjunct tool, providing a non-invasive, indirect assessment of bone microarchitecture derived from standard DXA images [3,4].

Children with Crohn’s disease, ulcerative colitis, and celiac disease are all at increased risk of impaired bone health due to shared disease mechanisms, including chronic inflammation, nutritional deficiencies, impaired growth, and delayed pubertal development.

The aim of the present study was to evaluate bone mass and TBS in children with autoimmune gastrointestinal diseases and to assess the potential clinical utility of TBS in this population. Specifically, we compared bone quantity (as assessed by BMD) and bone quality (as assessed by TBS) among the group of children with autoimmune gastrointestinal diseases, those with a previous history of bone fractures, and healthy controls. In addition, we investigated the relationships between TBS and selected anthropometric parameters, densitometric indices, and serum 25-hydroxyvitamin D [25(OH)D] levels.

## 2. Materials and Methods

Approval for the study was obtained from the Bioethical Committee at the Polish Mother’s Memorial Hospital—Research Institute (PMMH-RI) in Lodz, Poland.

This retrospective study included 152 children (mean age: 13.15 ± 3.24 years; 61 girls and 91 boys) aged 5–18 years. The study population consisted of patients from the Department of Endocrinology and Metabolic Diseases, as well as from the Pediatric Osteoporosis Outpatient Clinic and the Pediatric Endocrinology Outpatient Clinic all located at the Polish Mother’s Memorial Hospital—Research Institute in Lodz, Poland, who required specialist consultation between 2021 and 2025. Participants were divided into three groups: children with autoimmune gastrointestinal diseases (*n* = 45; including ulcerative colitis *n* = 23, Crohn’s disease *n* = 11, and celiac disease n = 11), children with a history of at least one bone fracture (*n* = 37) including long-bone fractures (*n* = 33) and vertebral fractures (*n* = 4), and a control group without bone fractures or autoimmune gastrointestinal diseases (*n* = 70).

Although Crohn’s disease, ulcerative colitis, and celiac disease differ in their underlying pathophysiology and treatment, they were analyzed together in the primary analyses because all represent chronic autoimmune gastrointestinal diseases associated with impaired bone health through shared mechanisms, including chronic inflammation, nutritional deficiencies, growth disturbances, and reduced bone mineral accrual. Disease-specific subgroup analyses were performed as secondary exploratory analyses to evaluate potential differences between individual disorders.

The fracture group included children with a history of one or more fractures. Owing to the retrospective design of the study, detailed information regarding the mechanism of injury, the exact number of fractures per patient, and the time elapsed since fracture occurrence was not consistently available for all participants. To minimize the potential influence of recent fractures on bone measurements, children who had sustained a fracture within the preceding 6 months were excluded from the study.

The control group consisted of children who had been referred to the pediatric endocrinology outpatient clinic, mainly for evaluation of short stature. Endocrine disorders and other chronic diseases affecting bone metabolism were excluded before study inclusion. Clinical data collected for each participant included the primary diagnosis, height, body weight, and pubertal stage assessed according to the Tanner scale [16]. For statistical analyses, pubertal development was categorized into three groups: Tanner stage I (prepubertal), Tanner stages II–III (early/mid-puberty), and Tanner stages IV–V (late puberty). Based on these parameters, the height standard deviation score (HSDS) and body weight standard deviation score (weight SDS) were calculated. Detailed characteristics of the study population are presented in Table 1. Additionally, body mass index (BMI) and BMI SDS values were determined using reference percentile charts for the Polish pediatric population [17]. Serum 25-hydroxyvitamin D [25(OH)D] concentrations were measured in all patients using an electrochemiluminescence immunoassay (ECLIA) with the Elecsys Vitamin D assay on the Cobas E 801 analyzer platform (Roche Diagnostics, Mannheim, Germany). Information regarding vitamin D supplementation was obtained from the medical records and included all participants. The season of blood sampling was determined from the date of examination and categorized as winter (December–February), spring (March–May), summer (June–August), or autumn (September–November). Information regarding calcium supplementation was not systematically collected and therefore was not evaluated.

DXA measurements were performed using the Hologic Horizon Bone Densitometry System (Hologic Inc., Marlborough, MA, USA), software version 007 and TBS analysis was retrieved from TBS iNsight software, version 3.0.3.0 (Medimaps Group SA, Geneva, Switzerland). For each patient, DXA assessments were obtained at two skeletal sites: total body less head (TBLH) and the lumbar spine (LS). To minimize the influence of anthropometric variability on lumbar spine DXA measurements, the bone mineral apparent density (BMAD) was calculated using the following formula: BMAD = Bone Mineral Content (BMC)/Bone Area (BA)^1.5 [18].

We employed an alternative normalization method for both projections to optimize the BMD Z-score. This method, which simultaneously accounts for both height and age, involves adjusting for the height-for-age Z-score (HAZ) [19,20]. Adjusted BMD Z-scores for age were calculated for both the Spine and TBLH projections. TBS values were normalized for gender and pubertal stage and expressed as TBS Z-scores according to the standards established in the publication by Kalkwarf et al. [6].

The inclusion criteria were as follows: age between 5 and 18 years; in the autoimmune gastrointestinal disease group, a confirmed diagnosis of Crohn’s disease, ulcerative colitis, or celiac disease; in the bone fracture group, a history of at least one long-bone or vertebral fracture; and completion of a DXA examination. The exclusion criteria included age below 5 years or above 18 years, the presence of comorbid conditions, and the use of medications known to significantly affect bone mineralization in children, fracture occurrence in the last 6 months.

Statistical analysis was performed using STATISTICA software, version 13.3 (StatSoft, Cracow, Poland). The Shapiro–Wilk test was used to assess the normality of data distribution, while Levene’s test was applied to evaluate the homogeneity of variances. For comparisons involving more than two groups, one-way analysis of variance (ANOVA) was used for variables with a normal distribution and homogeneous variances, whereas the Kruskal–Wallis test was applied for variables that did not meet these assumptions. When the overall comparison was statistically significant, pairwise comparisons between groups were subsequently performed using Student’s *t*-test or the Mann–Whitney U test, as appropriate. Correlations between variables were assessed using Pearson’s or Spearman’s correlation coefficients, depending on data distribution. To account for multiple comparisons in the correlation analyses, *p*-values were additionally adjusted using the Benjamini–Hochberg false discovery rate (FDR) procedure. Missing data were minimal and were handled using complete-case analysis. Descriptive statistics included the number of patients in each study group, as well as mean ± standard deviation (SD) and median with interquartile range (IQR; Q1–Q3) for continuous variables. A *p*-value < 0.05 was considered statistically significant. To identify factors independently associated with TBS, multivariable linear regression analyses were additionally performed. Prior to model construction, multicollinearity among candidate predictors was assessed using variance inflation factors (VIFs). Because TBS Z-score is already standardized for sex and pubertal stage, two separate regression models were constructed. The first model, with absolute TBS as the dependent variable, included pubertal stage, sex, BMI SDS, height SDS, serum 25(OH)D concentration, and study group as independent variables. The second model, with TBS Z-score as the dependent variable, included study group, BMI SDS, height SDS, and serum 25(OH)D concentration. Regression coefficients (B), 95% confidence intervals, adjusted R^2^ values, and *p*-values were reported. Effect sizes were calculated to complement significance testing. For comparisons involving one-way ANOVA, omega squared (ω^2^) was reported, whereas epsilon squared (ε^2^) was used for Kruskal–Wallis analyses. For pairwise comparisons performed using the Mann–Whitney U test, rank-biserial correlation (rrb) was calculated. Ninety-five percent confidence intervals for effect-size estimates were obtained using bootstrap resampling. Effect sizes were interpreted according to conventional thresholds and were used to facilitate assessment of the clinical relevance of the observed differences. Correlation analyses and disease-specific subgroup analyses were considered exploratory and were primarily intended to identify potential associations, whereas the multivariable regression analyses were performed to assess independent predictors of TBS.

## 3. Results

### 3.1. Analysis of Bone Mineralization Disorders and Trabecular Bone Score in Individual Study Groups

For the entire study population of 152 children, low bone mass (aBMD_for age_ Z-score ≤ −2) was observed in 30.3% of patients at the TBLH site and in 11.1% at the Spine. After adjustment for height (HAZ-adjusted aBMD Z-score), the prevalence of low bone mass decreased to 19.1% for TBLH and to 5.2% for Spine. No significant differences in aBMD_HAZ_ Z-score TBLH were observed among the study groups. However, when it comes to the analysis of the Spine projection, children with a history of bone fractures demonstrated significantly lower aBMD_HAZ_ Z-score Spine compared with both the autoimmune gastrointestinal disease group and the control group (Table 2).

In the entire analyzed cohort, a TBS Z-score ≤ −2 was identified in 5.2% of children, values between −2 and −1 in 20.4%, and values > −1 in 74.3% of participants. No significant differences in TBS or TBS Z-scores were observed among the study groups (Table 2). These findings refer to the unadjusted between-group comparisons. The results of the multivariable regression analyses are presented separately in Section 3.5.

**Figure 1:**
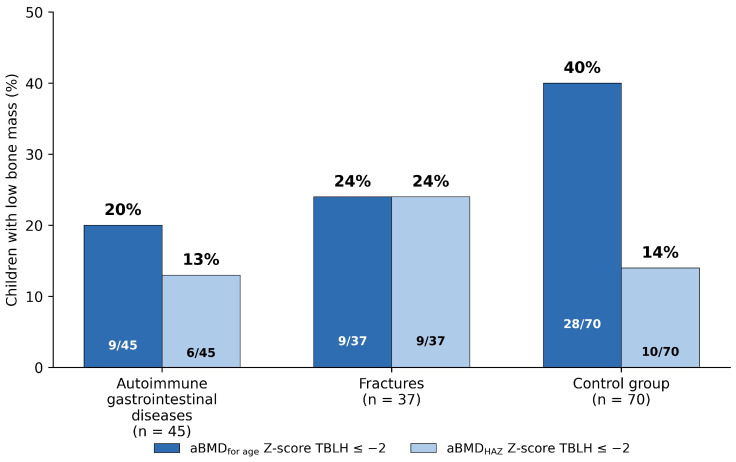
Prevalence of low bone mass in the study groups at TBLH site before and after height adjustment (aBMD_for age_ Z-score TBLH vs. aBMD_HAZ_ Z-score TBLH).

**Figure 2:**
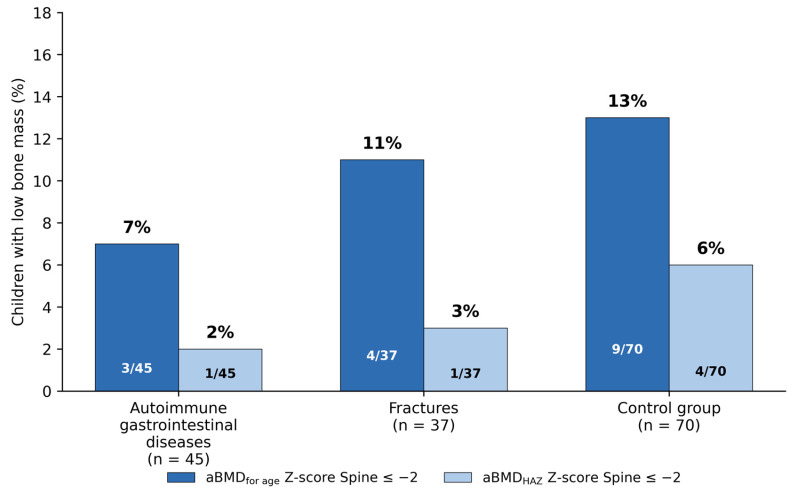
Prevalence of low bone mass in the study groups at the Spine site before and after height adjustment (aBMD_for age_ Z-score Spine vs. aBMD_HAZ_ Z-score Spine).

Figure 1 and Figure 2 present the prevalence of low bone mass at the TBLH and Spine sites across the individual study groups.

Among subjects with a TBS Z-score ≤ −2, low bone mass (aBMD_for age_ Z-score Spine ≤ −2) was found in 12.5% of participants, whereas among those with a TBS Z-score between −2 and −1, low bone mass was identified in 29%. In the TBLH projection, among participants with a TBS Z-score ≤ −2, low bone mass (aBMD_for age_ Z-score TBLH ≤ −2) was identified in 37.5% of subjects. Similarly, among those with a TBS Z-score between −2 and −1, low bone mass was found in 38.7% of subjects.

After adjustment of bone mineral density for height, low bone mass in the lumbar spine projection (aBMD_HAZ_ Spine ≤ −2) was not observed in any participant with a TBS Z-score ≤ −2. In contrast, among subjects with a TBS Z-score between −2 and −1, low bone mass was identified in 9.6% of participants. Following height adjustment, low bone mass in the TBLH projection (aBMD_HAZ_ TBLH ≤ −2) was observed in 25.0% of subjects with a TBS Z-score ≤ −2. Among participants with a TBS Z-score between −2 and −1, low bone mass remained present in 38.7% of subjects.

### 3.2. Comparison of Age, Anthropometric Parameters, DXA Parameters, and Serum 25(OH)D Levels Among Patient Groups According to Diagnosis

Patients with autoimmune gastrointestinal diseases differed significantly in terms of height expressed as HSDS compared to the control group (*p* = 0.002) (Table 1). The studied groups did not differ significantly in terms of age, body weight SDS, BMI SDS, or serum 25(OH)D concentration.

Statistical analysis revealed significant differences in HSDS between the control group and the clinical groups (patients with fractures and those with autoimmune gastrointestinal diseases). The control group exhibited significantly lower HSDS values because many of these children had been referred to the pediatric endocrinology outpatient clinic for evaluation of short stature, although endocrine causes of growth impairment had been excluded. Consequently, this group should not be considered representative of a healthy population. This recruitment strategy likely contributed to the relatively high prevalence of low bone mass observed in the control group before height adjustment. To minimize the confounding effect of stature on DXA-derived measurements, bone mineral density was additionally expressed as HAZ-adjusted aBMD Z-scores. These adjusted values were used throughout the analyses to reduce the influence of body size on the assessment of bone mineralization.

In the overall study cohort, vitamin D deficiency (25(OH)D < 30 ng/mL) was identified in 39.5% of participants. Only one child (0.65%) was found to have severe vitamin D deficiency (25(OH)D < 10 ng/mL). In 22 participants (14.5%), serum 25(OH)D concentrations ranged between 10 and 20 ng/mL, including 5 children (11.1%) with inflammatory bowel disease (IBD), 3 children (8.1%) with fractures, and 14 children (20.0%) in the control group. In 25.0% of all participants, serum 25(OH)D concentrations were between 20 and 30 ng/mL, including 11 children with IBD (24.4%), 9 children with fractures (24.3%), and 18 controls (25.7%).

Vitamin D supplementation was reported in 112 of 152 participants (73.7%). The proportion of supplemented participants differed significantly between the study groups: 44 of 45 children (97.8%) in the autoimmune gastrointestinal disease group, 32 of 37 (86.5%) in the fracture group, and 36 of 70 (51.4%) in the control group (χ^2^ = 34.48, *p* < 0.001). Serum 25(OH)D concentrations were significantly higher in supplemented than in non-supplemented participants (median 35.2 [IQR 29.3–41.5] vs. 23.1 [19.0–31.5] ng/mL; *p* < 0.001). The seasonal distribution of blood sampling did not differ significantly between the study groups (*p* = 0.556), and no significant seasonal differences were observed in serum 25(OH)D concentrations, TBS, TBS Z-scores, or height-adjusted BMD Z-scores.

DXA parameters, including TBS and TBS Z-score, in the individual study groups are presented in Table 2.

**Figure 3:**
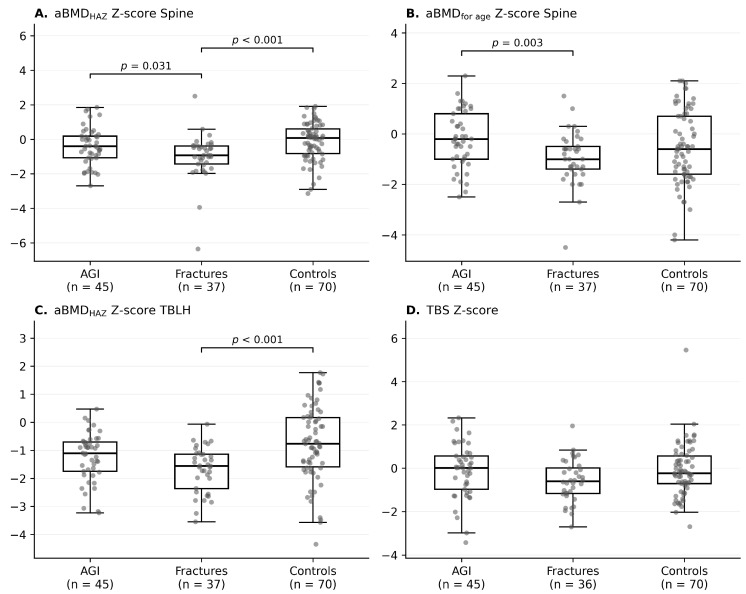
Comparison of aBMD_HAZ_ Z-score Spine (**A**), aBMD_for age_ Z-score Spine (**B**), aBMD_HAZ_ Z-score TBLH (**C**), and TBS Z-score (**D**) among study groups (children with fractures, healthy controls, and children with autoimmune gastrointestinal diseases—AGI diseases).

Children with a history of bone fractures had a significantly lower aBMD_HAZ_ Z-score Spine compared with the other groups (Figure 3A).

In contrast, the autoimmune gastrointestinal disease group demonstrated a significantly higher aBMD_HAZ_ Z-score Spine compared with the bone fracture group, while showing comparable values to healthy controls. In this group, unadjusted lumbar spine aBMD_for age_ Z-scores (not corrected for height) were also significantly higher than in the fracture group (Figure 3B).

In the fracture group, a significantly lower aBMD_HAZ_ Z-score TBLH was also observed compared with the healthy control group. However, no significant differences in this parameter were found between the autoimmune gastrointestinal disease group and either of the other two groups (Figure 3C).

No statistically significant differences were observed between the study groups in BMC Spine, aBMD Spine, BMAD, aBMD TBLH, aBMD_for age_ Z-score TBLH, BMC TBLH, TBS, or TBS Z-score (Figure 3D).

To complement hypothesis testing, effect sizes were calculated for the main between-group comparisons. Moderate between-group effects were observed for height SDS (ε^2^ = 0.101, 95% CI: 0.024–0.226), aBMD_HAZ_ Z-score Spine (ε^2^ = 0.087, 95% CI: 0.023–0.192), and aBMD_HAZ_ Z-score TBLH (ε^2^ = 0.104, 95% CI: 0.032–0.223). In contrast, effect sizes for absolute TBS (ω^2^ = 0.025, 95% CI: 0.000–0.114) and TBS Z-score (ε^2^ = 0.023, 95% CI: 0.000–0.111) were small, supporting the absence of clinically meaningful differences between study groups despite numerical variation.

### 3.3. Trabecular Bone Score by Pubertal Stage

In the study population, 21.7% (33/152) of children were prepubertal (Tanner stage I), 24.3% (37/152) were in Tanner stages II–III, and 53.3% (81/152) were in Tanner stages IV–V.

Children with advanced pubertal development (Tanner stages IV–V) demonstrated significantly higher TBS values compared with both prepubertal children (Tanner stage I; *p* = 0.00003) and children in Tanner stages II–III (*p* = 0.0002). Similarly, TBS Z-scores were significantly higher in children at Tanner stages IV–V than in prepubertal children (*p* = 0.00004) and those at Tanner stages II–III (*p* = 0.028).

When TBS values were analyzed separately by gender, a statistically significant difference was observed only between subjects in Tanner stages IV–V and those in Tanner stages II–III.

### 3.4. The Correlation Between TBS and TBS Z-Score and: Age, Anthropometric Parameters, DXA Parameters, and Serum 25(OH)D Concentration

Although, in the entire cohort, TBS correlated significantly with age, height SDS, body weight SDS, BMI SDS, and all DXA-derived parameters, TBS Z-scores showed positive correlations only with: aBMD Z-scores and HAZ-adjusted aBMD values at both measurement sites (TBLH and Spine), as well as with age (Table 3). No statistically significant associations were found between TBS Z-scores and height SDS, body weight SDS, or BMI SDS.

No significant association was observed between TBS parameters and serum 25(OH)D concentrations. In the entire study cohort, patients with normal serum 25(OH)D levels (>30 ng/mL; 91/152) did not differ significantly in terms of TBS or TBS Z-scores compared with children with vitamin D deficiency (25(OH)D ≤ 30 ng/mL) (61/152). Separate analyses of the individual study groups also did not reveal this association.

In the cohort of patients with autoimmune gastrointestinal diseases, TBS showed statistically significant positive correlations (*p* < 0.05) with age (r = 0.48), height SDS (r = 0.42), aBMD Spine (r = 0.60), BMAD (r = 0.39), BMC Spine (r = 0.67), BMC TBLH (r = 0.67), and aBMD TBLH (r = 0.64). TBS Z-score was also significantly correlated (*p* < 0.05) with height SDS (r = 0.33), lumbar spine aBMD_for-age_ Z-score (r = 0.36), aBMD_for-age_ Z-score TBLH (r = 0.51), and aBMD_HAZ_ Z-score TBLH (r = 0.39).

In the subgroup of patients with fractures, TBS also demonstrated statistically significant positive correlations (*p* < 0.05) with age (r = 0.68), BMC Spine (r = 0.81), BMD Spine (r = 0.80), aBMD_for-age_ Z-score Spine (r = 0.40), BMAD (r = 0.81), TBLH BMC (r = 0.71), and TBLH aBMD (r = 0.72). However, in this subgroup, no statistically significant associations were found between the assessed anthropometric parameters or DXA-derived measurements and TBS Z-score.

After adjustment for multiple testing using the Benjamini–Hochberg false discovery rate procedure, the correlations between absolute TBS and age, BMI SDS, height SDS, height-for-age Z-score, and BMAD remained statistically significant. For TBS Z-score, only the associations with height-adjusted aBMD Z-scores remained significant after correction.

In the overall study population, 8.5% of children were undernourished (BMI SDS ≤ −2), 65.8% had normal body weight (BMI SDS between −2 and 2), and 25.7% were classified as obese. Among the undernourished participants, four had autoimmune inflammatory bowel disease, four had a history of fractures, and five were controls. The obese subgroup included 6 (15.4%) children with autoimmune gastrointestinal diseases, 12 (30.8%) children with fractures, and 21 (53.8%) controls. In children with obesity (BMI SDS ≥ 2), TBS and TBS Z-scores did not differ significantly compared with children with normal BMI or those with BMI SDS ≤ −2 (*p* > 0.05).

### 3.5. Multivariable Analysis of Factors Associated with TBS

The results of the multivariable linear regression analyses are presented in Table 4. In the model using absolute TBS as the dependent variable, late pubertal stage (Tanner stages IV–V vs. Tanner stage I) was independently associated with higher TBS values (B = 0.073, 95% CI: 0.032–0.113, *p* = 0.001). In contrast, after adjustment for pubertal stage, sex, BMI SDS, height SDS, serum 25(OH)D concentration and study group, the children with a history of fractures had significantly lower absolute TBS values than those with autoimmune gastrointestinal diseases (B = −0.053, 95% CI: −0.094 to −0.012, *p* = 0.013), despite the absence of significant between-group differences in the unadjusted analyses. Tanner stages II–III, sex, BMI SDS, height SDS, serum 25(OH)D concentration, and membership in the control group were not independently associated with absolute TBS. The model explained 31.3% of the variability in absolute TBS (R^2^ = 0.313; adjusted R^2^ = 0.274; overall model *p* < 0.001).

Because the TBS Z-score is standardized for sex and pubertal stage, these variables were not included in the second regression model. In the model using TBS Z-score as the dependent variable, study group, BMI SDS, height SDS, and serum 25(OH)D concentration were not independently associated with TBS Z-score. Although children with fractures tended to have lower TBS Z-scores than children with autoimmune gastrointestinal diseases, this association did not reach statistical significance (B = −0.486, 95% CI: −1.017 to 0.045, *p* = 0.073). The explanatory power of this model was low (R^2^ = 0.031; adjusted R^2^ = −0.002; overall model *p* = 0.462).

### 3.6. Analysis of TBS and TBS Z-Score in Patients with Autoimmune Gastrointestinal Diseases

Additionally, a more detailed subgroup analysis was performed in patients with autoimmune gastrointestinal diseases. The detailed characteristics of this study group are presented in Table 5.

In the group of children with autoimmune gastrointestinal diseases, a TBS Z-score ≤ −2 was observed in 4/45 (8.9%) patients, while a TBS Z-score between −2 and −1 was found in 7/45 (15.5%) patients. In the same group, low bone mass at the lumbar spine (aBMD_for age_ Z-score Spine ≤ −2) was identified in 2/45 (4.4%) patients, and in 9/45 (20.0%) patients in the TBLH region. After adjustment for height (HAZ), these proportions were 2/45 (4.4%) for the spine and 8/45 (17.8%) for TBLH, respectively.

In children with celiac disease, no cases of TBS Z-score ≤ −2 were observed. However, low bone mass was identified in 1/11 (9.1%) patients at the lumbar spine (HAZ Spine) and in 4/11 (36.4%) patients at TBLH (HAZ TBLH).

Among children with Crohn’s disease, reduced TBS Z-score (≤−2) was observed in 2/11 (18.2%) patients, while no cases of concomitant low bone mass were identified in this subgroup.

In patients with ulcerative colitis, reduced TBS Z-score was observed in 2/23 (8.7%) patients. Low bone mass was identified in 1/23 (4.3%) patients at the lumbar spine (HAZ Spine) and in 4/23 (17.4%) patients at TBLH (HAZ TBLH).

The distribution of patients with reduced TBS and decreased BMD among the individual groups of patients with autoimmune gastrointestinal diseases is presented in Table 6. Notably, among the four patients with a reduced TBS Z-score, only one patient with ulcerative colitis exhibited concomitant low bone mass in the TBLH assessment. Two patients (one with ulcerative colitis and one with Crohn’s disease) had aBMD_HAZ_ Z-score TBLH values between −2 and −1, while the remaining patient had a TBLH aBMD_HAZ_ Z-score TBLH value > −1, consistent with normal bone mass. None of these four patients demonstrated impaired bone mineralization at the lumbar spine, with Spine aBMD_HAZ_ values ≥ −1 in all cases.

When analyzing only the group of patients with autoimmune gastrointestinal dis-eases, children with Crohn’s disease had a statistically significantly lower TBS compared with those with ulcerative colitis (*p* = 0.027). No significant difference was observed for the TBS Z-score.

Patients diagnosed with celiac disease, Crohn’s disease, and ulcerative colitis did not differ significantly (*p* > 0.05) in DXA-derived bone mass parameters, including lumbar spine aBMD_for age_ Z-score Spine, aBMD_HAZ_ Z-score Spine, aBMD_for age_ Z-score TBLH, and aBMD_HAZ_ Z-score TBLH.

## 4. Discussion

The present study evaluated TBS in children with autoimmune gastrointestinal diseases and compared its performance with conventional DXA-derived measures of bone mineral density. In our cohort, low bone mass (aBMD_for age_ Z-score ≤ −2) was relatively common, affecting 30.3% of patients in the TBLH assessment and 11.1% in the lumbar spine assessment. By comparison, significantly impaired trabecular microarchitecture, defined as a TBS Z-score ≤ −2, was identified in only 5.2% of participants. These results indicate that, in children with autoimmune gastrointestinal diseases, abnormalities in bone quantity may be more prevalent—or more readily detected—than alterations in bone quality when evaluated using currently available pediatric TBS algorithms.

Limited concordance between TBS and aBMD has also been reported in healthy pediatric populations. Kalkwarf et al. demonstrated that only approximately 30% of children with a low TBS Z-score also had low aBMD, while TBS accounted for only a modest proportion of the variability in Spine aBMD and BMAD Z-scores [6]. Similar findings in our cohort suggest that TBS captures skeletal characteristics that differ, at least in part, from those reflected by aBMD. However, alternative explanations should also be taken into consideration. TBS measurements in children are characterized by lower precision than DXA-derived aBMD, pediatric reference standards remain relatively recent, and differences between available TBS algorithms may additionally contribute to discrepancies between TBS and aBMD classifications [21]. Therefore, the present findings should not be interpreted as definitive evidence that TBS reflects an entirely independent aspect of skeletal quality, but rather as confirmation that the relationship between bone density and bone microarchitecture in growing children remains complex and incompletely understood.

This may be explained by the associations observed between TBS, TBS Z-scores, age, and anthropometric parameters. In the overall cohort, absolute TBS demonstrated a significant positive correlation with chronological age. Similar findings have been reported by other authors [22,23]. This age-related increase likely reflects the physiological maturation of trabecular microarchitecture occurring throughout growth, particularly during the pubertal transition. Within the total study population, absolute TBS showed positive correlations with height SDS, body weight SDS, and BMI SDS. In contrast, TBS Z-scores were not significantly correlated with height SDS, weight SDS, or BMI SDS. This absence of association suggests that, in the general cohort, TBS Z-scores constitute a robust indicator of bone quality that is not materially influenced by body size [6]. When the analysis was restricted to the autoimmune gastrointestinal disease subgroup, however, a significant positive correlation emerged between TBS Z-score and height SDS (r = 0.33, *p* < 0.05). This finding indicates that, in children with chronic gastrointestinal disease, impaired linear growth—reflected by lower height SDS—may be more closely linked to deterioration of trabecular bone microarchitecture [24]. Furthermore, the study revealed moderate-to-strong correlations between TBS and conventional DXA-derived parameters, while clearly distinguishing between bone quantity and bone quality. Absolute TBS correlated strongly with lumbar spine aBMD and BMC. In addition, TBS Z-scores were significantly associated with aBMD_for age_ Z-scores and aBMD_HAZ_ Z-scores. These moderate correlations of Z-scores are consistent with the observations reported by Kalkwarf et al. [6].

Multivariable analyses further clarified these relationships. After adjustment for pubertal stage, sex, BMI SDS, height SDS, serum 25(OH)D concentration among the study group, only advanced pubertal stage and fracture history remained independently associated with absolute TBS, whereas no independent predictors of TBS Z-score were identified. These findings are consistent with previous pediatric studies demonstrating that absolute TBS is strongly influenced by skeletal maturation and developmental status, whereas normalization substantially reduces the influence of growth-related factors on TBS values [6,21,22,23] Consistent with these observations, effect-size analyses manifested only small between-group differences for both absolute TBS and TBS Z-score, whereas moderate effects were observed for height-adjusted DXA-derived BMD parameters. Together, these findings suggest that, in the present cohort, conventional DXA-derived indices appeared more sensitive than TBS for detecting between-group differences in skeletal status.

Our findings further confirm a strong association between TBS and pubertal development. Significantly higher TBS and TBS Z-scores were observed in children at Tanner stages IV–V compared with those in prepubertal or early pubertal stages. This is consistent with evidence indicating that TBS increases predominantly during the pubertal growth spurt, while remaining relatively stable both before its onset and after its completion [6]. Previous studies reported that TBS values remain largely unchanged until approximately 9 years of age in girls and 10 years of age in boys, corresponding to pubertal onset. Thereafter, a more pronounced increase is observed in girls, beginning around age 9 and reaching a peak at approximately 16 years, whereas boys exhibit a more gradual rise starting at 10–11 years and peaking around 18 years. In addition, marked differences across Tanner stages have been described, with the most substantial changes occurring between stages II and III [7]. The correlations observed between TBS, chronological age, and anthropometric parameters therefore appear to reflect normal skeletal maturation rather than disease-specific effects.

Patients with autoimmune gastrointestinal diseases, particularly those with Crohn’s disease, are at increased risk of impaired bone accrual due to chronic systemic inflammation, malnutrition, and glucocorticoid exposure [2,9,14]. In the present analysis of the gastrointestinal subgroup, children with Crohn’s disease exhibited significantly lower TBS values compared with those diagnosed with ulcerative colitis. Comparable findings were reported by Levy-Shraga et al., who demonstrated lower mean TBS values in patients with Crohn’s disease than in those with ulcerative colitis [25]. However, our finding should be interpreted cautiously, as the comparison involved a limited number of patients, no significant differences were observed for TBS Z-scores, and the disease subgroup analyses included clinically heterogeneous disorders with different mechanisms affecting bone metabolism. Consequently, these observations should be regarded as hypothesis-generating and require confirmation in larger disease-specific cohorts.

Interestingly, despite the frequent reporting of aBMD deficits in pediatric intestinal bowel diseases, our study did not demonstrate significant differences in absolute TBS or TBS Z-scores between the autoimmune gastrointestinal disease, fracture, and control groups. This interpretation was further supported by the small effect sizes observed for both TBS parameters, whereas moderate effect sizes were demonstrated for height-adjusted DXA-derived bone mineral density. Together, these findings suggest that conventional DXA remained more sensitive than TBS in identifying skeletal differences within the present cohort. The absence of significant between-group differences in TBS may reflect limited statistical power, clinical heterogeneity of the autoimmune gastrointestinal disease group, or the influence of unmeasured disease-related factors such as disease duration, inflammatory activity, nutritional status, glucocorticoid exposure, and remission status. It cannot be ruled out that the deterioration of trabecular microarchitecture develops later than decreasing bone mineral density during the course of these disorders. In a similar manner, Rehberg et al. reported comparable TBS values between children with cerebral palsy and healthy controls, suggesting that TBS may not fully capture the extent of skeletal fragility in certain high-risk pediatric populations [21]. It therefore cannot be excluded that, in the pediatric population, TBS may not represent a reliable indicator of fracture risk. This hypothesis—that bone mineral density, rather than bone microarchitecture and quality, may exert a greater influence on fracture susceptibility—finds support in the study by Nobile et al., who evaluated 216 participants aged 8–25 years [26]. In their cohort, low BMD was identified in 12.5% of patients at the lumbar spine and in 27% based on total body measurements, while the overall prevalence of lifetime fractures was 11.8%. Importantly, individuals with a history of fractures exhibited significantly lower spine BMD Z-scores and total body BMD Z-scores compared with those without fractures. Moreover, the prevalence of fractures was significantly higher among patients with a Spine BMD Z-score ≤ −2 SD than in those with values > −2 SD (28% vs. 10%; *p* = 0.015).

The role of vitamin D in maintaining skeletal integrity is well established, as it facilitates intestinal calcium absorption and bone mineralization. However, in the present study, no statistically significant correlation was observed between serum 25-hydroxyvitamin D [25(OH)D] levels and either absolute TBS or TBS Z-score. Comparable findings were reported by Shah et al., who demonstrated no significant associations between serum 25(OH)D concentrations and aBMD, trabecular density, or cortical porosity [27]. Importantly, the authors did not question the established relationship between severe vitamin D deficiency and osteomalacia; however, they emphasized that the threshold duration and severity of 25(OH)D deficiency required to induce measurable changes in aBMD and bone microstructure remain uncertain [27].

In the present cohort, vitamin D supplementation was significantly more frequent among children with autoimmune gastrointestinal diseases and those with a history of fractures than in the control group, and supplemented participants had significantly higher serum 25(OH)D concentrations. Therefore, vitamin D supplementation should be considered a potential confounding factor when interpreting the observed vitamin D status. These findings are in line with previous studies indicating that correction of vitamin D deficiency through supplementation may improve bone mineral status in children, although its effect on bone quality parameters remains less well established [27]. Nevertheless, the seasonal distribution of blood sampling was comparable across the study groups, making it unlikely that seasonal variation substantially influenced serum 25(OH)D concentrations or the observed skeletal outcomes.

Although TBS is a well-established predictor of fracture risk in adults, its utility as a predictive marker in pediatric populations remains under investigation [6]. In the present cohort, no significant differences in TBS were observed between children with a history of fractures and those without fractures in the unadjusted group comparisons. However, in the multivariable regression analysis adjusting for pubertal stage, sex, BMI SDS, height SDS, and serum 25(OH)D concentration, fracture history remained independently associated with lower absolute TBS, although no independent association was found for TBS Z-score. By contrast, children with a previous history of fractures demonstrated significantly lower aBMD_for age_ Z-score Spine, supporting the view that conventional DXA-derived bone mineral density remains the more informative measure of skeletal fragility in this population. These findings are consistent with previous pediatric studies providing an association between fracture history and reduced aBMD [26]. Nevertheless, because the observed association was identified only after multivariable adjustment and the study was cross-sectional, these findings should not be interpreted as evidence that TBS predicts fracture risk. Prospective longitudinal studies are required to determine whether TBS provides clinically meaningful information beyond conventional DXA in fracture risk assessment during childhood.

The present study has several limitations. First, its cross-sectional design allowed assessment of skeletal status at only a single time point and did not permit evaluation of longitudinal changes in bone mineral accrual or trabecular microarchitecture. Second, because of the retrospective design, detailed information regarding disease duration, inflammatory activity, cumulative glucocorticoid exposure, biologic therapy, nutritional status, calcium intake, physical activity, and remission status was not consistently available and therefore could not be incorporated into the analyses. These factors are widely acknowledged as key factors of bone health in children with inflammatory bowel disease and celiac disease and may have contributed to within-group heterogeneity.

Another important limitation relates to the composition of the autoimmune gastrointestinal disease cohort. Although these disorders share several mechanisms affecting skeletal health, they differ substantially in inflammatory burden, treatment strategies, and nutritional consequences. Consequently, the disease-specific subgroup analyses should be considered exploratory and interpreted cautiously until confirmed in larger prospective cohorts.

The control group also warrants consideration. Although participants had no history of fractures or autoimmune gastrointestinal disease, they were recruited from a tertiary pediatric endocrinology outpatient clinic rather than from the general pediatric population, and many had been referred because of short stature. While the use of height-adjusted BMD Z-scores reduced the influence of stature on DXA measurements, this recruitment strategy limits the generalizability of comparisons with a truly healthy reference population.

Finally, despite application of the Benjamini–Hochberg false discovery rate correction, the relatively large number of statistical comparisons increases the possibility of type I error. Accordingly, the exploratory findings should be interpreted with caution and require confirmation in adequately powered prospective studies.

## 5. Conclusions

In this cross-sectional study, neither absolute TBS nor TBS Z-scores differed significantly between children with autoimmune gastrointestinal diseases, children with a history of fractures, and controls in the unadjusted analyses. TBS was associated with selected DXA-derived measures of bone mineral density, and fracture history was independently associated with lower absolute TBS after multivariable adjustment, whereas no independent predictors of TBS Z-score were identified. Overall, these findings suggest that the clinical role of TBS in the assessment of pediatric bone health remains to be established. Larger prospective studies with adequately powered disease-specific cohorts and longitudinal fracture outcomes are required to determine whether TBS provides clinically meaningful information beyond conventional DXA in the assessment of bone health and fracture risk in children.

## Figures and Tables

**Table 1:** Comparison of anthropometric parameters and serum 25(OH)D levels according to patient diagnosis (* *p* < 0.05).

Diagnosis	Autoimmune Gastrointestinal Disease Mean ± SD Median (Q1–Q3)	Fractures Mean ± SD Median (Q1–Q3)	Control Group Mean ± SD Median (Q1–Q3)	Total Study Group
Gender	F: 17 (37.8%) M: 28 (62.2%)	F: 15 (40.5%) M: 22 (59.5%)	F: 29 (41.4%) M: 41 (58.6%)	F: 61 (40.1%) M: 91 (59.9%)
Age [years]	13.75 ± 3.43 15.34 (10.33–16.56)	12.83 ± 3.51 13.19 (10.89–15.78)	12.95 ± 2.94 13.45 (10.70–15.45)	13.16 ± 3.24 13.75 (10.67–15.87)
Height SDS	−0.05 ± 1.09 −0.33 (−0.91–1.00) *	−0.39 ± 1.37 −0.15 (−0.83–0.33)	−1.07 ± 1.96 −1.41 (−2.54–0.00) *	−0.60 ± 1.66 −0.62 (−1.73–0.42)
Body weight SDS	0.27 ± 1.61 0.00 (−0.83–0.75)	0.37 ± 2.18 −0.14 (−1.33–1.60)	0.51 ± 3.31 −0.74 (−2.00–1.71)	0.41 ± 2.63 −0.78 (−1.53–1.61)
BMI SDS	0.31 ± 1.96 0.03 (−0.88–1.29)	0.80 ± 2.63 0.46 (−1.15–2.36)	1.32 ± 3.53 0.01 (−1.45–2.71)	0.89 ± 2.94 0.04 (−1.19–2.13)
25(OH)D [ng/mL]	33.43 ± 9.57 35.20 (27.00–40.20)	35.69 ± 14.42 34.50 (29.20–41.50)	31.04 ± 12.52 31.30 (21.90–37.00)	32.88 ± 12.31 33.30 (23.55–39.30)

*—values marked with the same symbols in the same row differ significantly from each other in terms of the given *p* value.

**Table 2:** Comparison of DXA parameters across diagnostic groups (*, ^#^ *p* < 0.05).

Diagnosis	Autoimmune Gastrointestinal Disease Mean ± SD Median (Q1–Q3)	Fractures Mean ± SD Median (Q1–Q3)	Control Group Mean ± SD Median (Q1–Q3)	Total Study Group	*p*	Effect Size (95% CI)
BMC TBLH [g]	1242.76 ± 490.90 1288.77 (793.62–1573.92)	1064.59 ± 481.27 1077.15 (706.11–1336.65)	1077.38 ± 485.42 1030.63 (672.50–1379.24)	1123.23 ± 489.07 1054.58 (687.48–1498.04)	0.125	ε^2^ = 0.015 (0.000–0.096)
BMD TBLH [g/cm^2^]	0.80 ± 0.13 0.83 (0.70–0.88)	0.73 ± 0.14 0.74 (0.63–0.84)	0.76 ± 0.15 0.76 (0.63–0.86)	0.76 ± 0.15 0.77 (0.64–0.88)	0.102	ω^2^ = 0.017 (0.000–0.093)
aBMD_for age_ Z-score TBLH	−1.22 ± 1.09 −1.10 (1.70–(−0.60))	−1.76 ± 1.17 −1.50 (−1.90–(−1.30))	−1.34 ± 1.82 −1.65 (−2.50–0.20)	−1.13 ± 1.15 −1.12 (−1.77–(−0.58))	0.092	ε^2^ = 0.019 (0.000–0.101)
aBMD_HAZ_ Z-score TBLH	−1.23 ± 0.87 −1.10 (−1.75–(−0.70))	−1.69 ± 0.81 −1.56 (−2.36–(−1.14)) *	−0.76 ± 1.33 −0.76 (−1.63–0.17) *	−1.38 ± 1.50 −1.35 (−2.20–(−0.40))	<0.001	ε^2^ = 0.104 (0.032–0.223)
BMC Spine [g]	43.03 ± 17.80 44.72 (25.63–53.73)	35.04 ± 16.70 34.46 (18.10–45.16)	35.39 ± 16.98 32.00 (22.14–46.88)	37.57 ± 17.41 35.45 (23.09–49.05)	0.038	ε^2^ = 0.031 (0.000–0.108)
BMD Spine [g/cm^2^]	0.80 ± 0.17 0.80 (0.65–0.90)	0.70 ± 0.19 0.72 (0.54–0.81)	0.73 ± 0.20 0.70 (0.55–0.85)	0.74 ± 0.19 0.73 (0.58–0.86)	0.027	ε^2^ = 0.035 (0.000–0.115)
BMAD	0.11 ± 0.01 0.11 (0.10–0.12)	0.10 ± 0.02 0.10 (0.09–0.11)	0.11 ± 0.02 0.10 (0.09–1.12)	0.11 ± 0.02 0.11 (0.09–1.12)	0.041	ε^2^ = 0.029 (0.000–0.107)
aBMD_for age_ Z-score Spine	−0.21 ± 1.12 −0.20 (−1.00–0.80) *	−0.95 ± 1.04 −1.00 (−1.40–(−0.50)) *	−0.55 ± 1.46 −0.60 (−1.60–0.70)	−0.55 ± 1.46 −0.60 (−1.60–0.70)	0.027	ε^2^ = 0.035 (0.000–0.119)
aBMD_HAZ_ Z-score Spine	−0.38 ± 1.08 −0.40 (−1.07–0.19) *	−1.00 ± 1.33 −0.92 (−1.43–(0.40)) * ^#^	−0.13 ± 1.09 0.08 (−0.85–0.63) ^#^	−0.42 ± 1.29 −0.60 (−1.40–0.30)	<0.001	ε^2^ = 0.087 (0.023–0.192)
TBS	1.33 ± 0.11 1.31 (1.25–1.42)	1.27 1 ± 0.11 1.27 (1.18–1.37)	1.31 ± 0.10 1.31 (1.25–1.37)	1.31 ± 0.11 1.31 (1.24–1.38)	0.056	ω^2^ = 0.025 (0.000–0.114)
TBS Z-score	−0.11 ± 1.25 0.01 (−0.97–0.56)	−0.58 ± 0.97 −0.60 (−1.17–0.04)	−0.10 ± 1.19 −0.22 (−0.71–0.59)	−0.22 ± 1.18 −0.23 (−1.07–0.51)	0.069	ε^2^ = 0.023 (0.000–0.111)

*, ^#^—values marked with the same symbols in the same row differ significantly from each other in terms of the given *p* value. Effect sizes are expressed as omega squared (ω^2^) for one-way ANOVA and epsilon squared (ε^2^) for Kruskal–Wallis analyses. Ninety-five percent confidence intervals (95% CI) were obtained using bootstrap resampling.

**Table 3:** Correlations between TBS and TBS Z-score with chronological age, auxological parameters, serum 25(OH)D levels, and DXA parameters in the entire study population (* *p* < 0.05).

	TBS	TBS Z-Score
Age	0.48 *	−0.18 *
Height SDS	0.23 *	−0.02
Body weight SDS	0.26 *	−0.01
BMI SDS	0.20 *	0.02
25(OH)D	−0.07	−0.01
BMD Spine	0.64 *	0.006
BMC Spine	0.66 *	0.003
aBMD_for age_ Z-score Spine	0.33 *	0.25 *
aBMD_HAZ_ Z-score Spine	0.21 *	0.28 *
BMAD	0.53 *	0.04
BMD TBLH	0.64 *	0.01
BMC TBLH	0.63 *	0.002
aBMD_for age_ Z-score TBLH	0.31 *	0.25 *
aBMD_HAZ_ Z-score TBLH	0.21 *	0.31 *

*—values marked with the same symbols in the same row differ significantly from each other in terms of the given *p* value.

**Table 4:** Multivariable linear regression analyses of factors associated with absolute TBS and TBS Z-score among the studied subjects (*n* = 152).

Predictor	Absolute TBS B (95% CI)	*p*	TBS Z-Score B (95% CI)	*p*
**Tanner stage II–III vs. Tanner I**	−0.037 (−0.082 to 0.009)	0.112	—	—
**Tanner stage IV–V vs. Tanner I**	0.073 (0.032 to 0.113)	0.001	—	—
**Male sex**	0.010 (−0.022 to 0.042)	0.532	—	—
**BMI SDS**	0.004 (−0.002 to 0.009)	0.182	0.010 (−0.058 to 0.078)	0.766
**Height SDS**	0.009 (−0.002 to 0.021)	0.103	−0.000 (−0.141 to 0.141)	0.997
**25(OH)D (ng/mL)**	−0.000 (−0.001 to 0.001)	0.938	0.001 (−0.017 to 0.019)	0.888
**Control group vs. AGI**	−0.004 (−0.042 to 0.033)	0.825	0.006 (−0.474 to 0.485)	0.981
**Fracture group vs. AGI**	−0.053 (−0.094 to −0.012)	0.013	−0.486 (−1.017 to 0.045)	0.073
**Model Statistics**	**Absolute TBS**		**TBS Z-Score**	
**Number of participants**	152		152	
**R** ^2^	0.313		0.031	
**Adjusted R** ^2^	0.274		−0.002	
**F statistic**	8.03		0.93	
**Overall model *p***	<0.001		0.462	

Abbreviations: AGI, autoimmune gastrointestinal diseases; BMI, body mass index; CI, confidence interval; SDS, standard deviation score; TBS, trabecular bone score. Reference categories: Tanner stage I (prepubertal), female sex, and the AGI group.

**Table 5:** Comparison of sex distribution, age, anthropometric parameters, TBS, and TBS Z-scores across subgroups of patients with autoimmune gastrointestinal diseases.

	Celiac Disease *N* = 11	Crohn Disease *N* = 11	Ulcerative Colitis *N* = 23
Gender	F: 3 (27.3%) M: 8 (72.7%)	F: 6 (54.5%) M: 5 (45.5%)	F: 8 (34.8%) M: 15 (65.2%)
Age [years]	12.48 ± 4.13 13.18 (7.50–16.57)	12.78 ± 3.48 12.41(9.84–16.51)	14.83 ± 2.80 15.87 (13.32–16.57)
Height SDS	−0.26 ± 1.299 −0.06 (−1.62–1.00)	−0.63 ± 0.47 −0.80 (−0.92–(−0.33)	0.33 ± 1.08 −0.14 (−0.36–1.17)
Body weight SDS	0.29 ± 1.67 −0.13 (−0.53–0.71)	0.46 ± 1.90 −0.29 (−1.00–1.47)	0.18 ± 1.51 0.00 (−0.93–1.78)
BMI SDS	0.58 ± 1.66 0.03 (−0.32–1.29)	1.05 ± 2.67 0.32 (−0.68–1.78)	1.05 ± 2.67 −0.17 (−0.16–1.18)
TBS	1.32 ± 0.08 1.31 (1.28–1.41)	1.27 ± 0.12 1.22 (1.17–1.42) *	1.37 ± 0.10 1.37 (1.30–1.45) *
TBS Z-score	−0.21 ± 1.05 −0.18 (−1.27–0.40)	−0.55 ± 1.43 −0.01 (−0.98–0.24)	0.14 ± 1.24 0.29 (−1.08–1.17)

*—values marked with the same symbols in the same row differ significantly from each other in terms of the given *p* value.

**Table 6:** Distribution of patients with reduced TBS and reduced bone mineral density according to type of autoimmune gastrointestinal disease.

	Celiac Disease *N* = 11	Crohn Disease *N* = 11	Ulcerative Colitis *N* = 23
TBS Z-score ≤ −2.0	-	2	2
aBMD_HAZ_ Z-score Spine ≤ −2.0	1	-	1
aBMD_HAZ_ Z-score TBLH ≤ −2.0	4	-	4
TBS Z-score > −2.0 but <−1.0	3	-	4
aBMD_HAZ_ Z-score Spine ≤ −2.0	4	1	4
aBMD_HAZ_ Z-score TBLH ≤ −2.0	2	3	10

## Data Availability

The data presented in this study are available on request from the corresponding author due to ethical and privacy restrictions.
